# How Does MBCT for Depression Work? Studying Cognitive and Affective Mediation Pathways

**DOI:** 10.1371/journal.pone.0072778

**Published:** 2013-08-23

**Authors:** Tim Batink, Frenk Peeters, Nicole Geschwind, Jim van Os, Marieke Wichers

**Affiliations:** 1 Department of Psychiatry and Psychology, Maastricht University Medical Centre, South Limburg Mental Health Research and Teaching Network, EURON, Maastricht, The Netherlands; 2 Department of Clinical Psychological Science, Maastricht University, Maastricht, The Netherlands; 3 Department of Psychosis Studies, Institute of Psychiatry, King’s College London, King’s Health Partners, London, England; University of Groningen, Netherlands

## Abstract

Mindfulness based cognitive therapy (MBCT) is a non-pharmacological intervention to reduce current symptoms and to prevent recurrence of major depressive disorder. At present, it is not well understood which underlying mechanisms during MBCT are associated with its efficacy. The current study (n = 130) was designed to examine the roles of mindfulness skills, rumination, worry and affect, and the interplay between those factors, in the mechanisms of change in MBCT for residual depressive symptoms. An exploratory but systematic approach was chosen using Sobel-Goodman mediation analyses to identify mediators on the pathway from MBCT to reduction in depressive symptoms. We replicated earlier findings that therapeutic effects of MBCT are mediated by changes in mindfulness skills and worry. Second, results showed that changes in momentary positive and negative affect significantly mediated the efficacy of MBCT, and also mediated the effect of worry on depressive symptoms. Third, within the group of patients with a prior history of ≤ 2 episodes of MDD, predominantly changes in cognitive and to a lesser extent affective processes mediated the effect of MBCT. However, within the group of patients with a prior history of ≥ 3 episodes of MDD, only changes in affect were significant mediators for the effect of MBCT.

Trail Registration: Nederlands Trial Register NTR1084

## Introduction

Major depressive disorder (MDD) is an invalidating condition, with high rates of residual symptoms and high probability of recurrence [1]. Segal, Williams & Teasdale originally developed Mindfulness Based Cognitive Therapy (MBCT, [2]) as a non-pharmacological intervention to prevent recurrence of MDD. MBCT is a group-based, 8-week training, consisting of meditation exercises combined with cognitive behavioural techniques. The focus of MBCT is to teach individuals to become more aware of thoughts and feelings and to relate to them in a wider, decentered perspective as "mental events" rather than as aspects of the self or as necessarily accurate reflections of reality. Being mindful is a skill that can be learned by doing regular exercises, to train to focus one’s attention on the present moment. It is assumed that the cultivation of a detached, decentered relationship to depression-related thoughts and feelings is central to the development of skills that prevent the escalation of negative thinking patterns at times of potential recurrence [3]. Although MBCT was originally developed as a preventive intervention, subsequent studies reported that MBCT also appeared effective as an intervention for the acute treatment phase in MDD, and for the reduction of residual symptomatology [4–9].

At present, it is not well understood how MBCT leads to reduction and prevention of symptoms of depression [10–12]. Understanding the mechanisms underlying change arguably is the first step in further optimizing any treatment. Therefore, an important initial step in establishing mechanisms of change in MBCT is to identify variables that mediate its therapeutic effects [13].

MBCT was designed to reduce the contribution of depressed cognitions (reactivated by negative affect) to relapse and recurrence by training the patients in applying Mindfulness-skills [14]. One of the few studies that provided empirical evidence for putative mediating factors in the efficacy of MBCT as a preventive treatment was published recently [15]. Consistent with MBCT’s theoretical premise, increases in mindfulness skills and self-compassion across treatment mediated the effect of MBCT on depressive relapse during follow-up. These initial results were confirmed in a subsequent study [16], that also reported that mindfulness skills and rumination mediated the effect of MBCT on reduction of depressive symptoms.

In an exploratory mediation analysis, van Aalderen and colleagues [9] found that the effectiveness of MBCT in reducing current levels of depression was mediated by a decrease in worry, rumination and an increase in the mindfulness skill ‘accept without judgment’. These results are in line with previous findings [15,16] but seem to be more specific in identifying which particular mindfulness skills mediate therapeutic effects. Another study in individuals with residual symptoms by Geschwind and colleagues [17] reported that the effect of MBCT was associated with increased experience of momentary positive emotions, greater appreciation of, and enhanced responsiveness to, pleasant daily-life activities. These changes appeared partially independent of more commonly observed decreases in worry and rumination. Together, this suggests that both changes in cognitive and affective processes contribute to reductions in depressive symptomatology. However, it is unknown how changes in mindfulness skills and cognition relate to these affective changes in bringing about clinical improvement during MBCT.

Further knowledge is also needed regarding the role of previous episodes of depression in relation to the efficacy of MBCT. Initial research on the efficacy of MBCT reported differential effects of MBCT on relapse prevention contingent on number of previous episodes. The impact on prevention of future episodes seemed restricted to patients with three or more depressive episodes, whereas no effect was observed in patients with two or fewer episodes [14,18]. Based on these early findings, subsequent research in the field of MBCT did not include participants with two or fewer previous episodes. However, a recent study [5] reported equal effectiveness of MBCT in reducing depressive symptoms for participants with few (≤2) or multiple (≥3) previous depressive episodes. This divergent finding may be explained by the difference in therapeutic goal: prevention of recurrence of depression versus reduction of current depressive symptomatology. It cannot be ruled out that MBCT, when applied for reduction of residual symptoms, exerts therapeutic effects through different mechanisms of change in participants with ≤2 previous depressive episodes compared to participants with ≥3 previous depressive episodes. Thus it could be hypothesized that, although the efficacy of MBCT in reducing residual symptoms may be independent from previous number of episodes, a difference in underlying mechanism of change may still result in differential risk for long-term course (recurrence) of MDD.

The current study was designed to examine the role of mindfulness skills, rumination/worry and affect in the mechanisms of change of MBCT in a sample with residual depressive symptoms. Participants meeting criteria for a full episode of major depressive disorder were excluded. Because affective states cannot be reliably measured retrospectively [19], the design of this study -of which data already have been reported previously [17]- used frequent and prospective in-the-moment measurements of affect, thus minimizing recall bias. Whereas the previous report [17] focused on change in positive affect and reward experience following MBCT, the current manuscript tries to identify the cognitive and affective mediators of the effect of MBCT on reduction of depressive symptoms.

The current analysis is building on, and extending the study of van Aalderen and co-workers [9]. The first aim was to replicate the results reported by these authors with regard to the question to what degree worry, rumination and mindfulness skills mediate the reduction of depressive symptoms in MBCT. A second aim was to examine how changes in negative and positive affect relate to changes in worry, rumination and mindfulness skills and to what extent affective changes mediate the effects of MBCT. Combining both cognitive and affective variables in the mediation analyses may provide a better understanding of the processes that mediate the effects of MBCT on depressive symptomatology. A final aim was to examine to what degree different mechanisms of change may underlie the impact of MBCT on symptom reduction in participants with multiple major depressive episodes (≥3 MDE) compared to those with fewer episodes (≤2 MDE).

## Methods

### Participants

**Figure 1:**
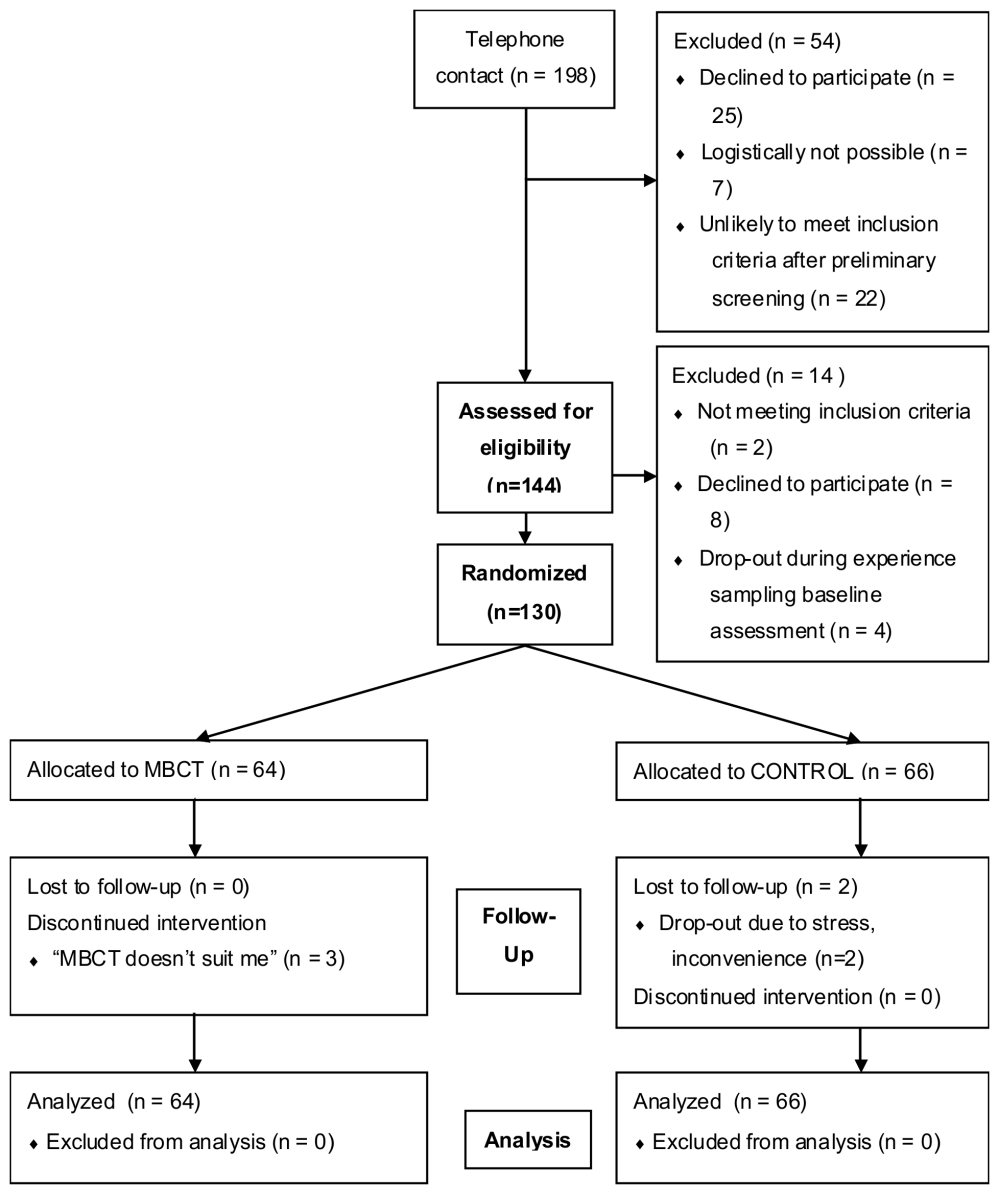
CONSORT participant flow diagram. MBCT = Mindfulness-based Cognitive Behavioral Therapy; CONTROL = waitlist control condition.

Figure 1 shows the flow of participants through the study. Adults with current residual depressive symptoms after at least one episode of major depressive disorder (mean age 43.9 years, SD 9.6; 75% female; all Caucasian) were randomized to MBCT (n=64) or treatment as usual (TAU; n=66) in a parallel, open-label, randomized controlled trial (MindMaastricht; trial number NTR1084, Netherlands Trial Register; http://www.trialregister.nl/trialreg/admin/rctview.asp?TC=1084). Participants were recruited from outpatient mental health care facilities in Maastricht and through posters in public spaces. Residual symptomatology was defined as a score of seven or higher on the 17-item Hamilton Depression Rating Scale (HDRS, [20]) at the time of screening. Exclusion criteria were fulfilling criteria for a current major depressive episode, a life-time diagnosis of schizophrenia, psychotic episodes in the past year, general conditions that made participation in a group intervention impossible, and recent (past four weeks) or upcoming changes in ongoing psychological or pharmacological treatment. Sample size (n ≥ 120) was determined on the basis of sufficient power for hypothesized gene-environment interactions on momentary assessment outcomes (not analyzed here). For more details on the study design see 17. The protocol for this trial and supporting CONSORT checklist are available as supporting information; see Checklist S1 and Protocol S1.

### Measures

#### Inventory of Depressive Symptoms (Self-Rating; IDS-SR^30^)

The IDS-SR^30^ [21] is a self-rating scale, which includes 30 items rated 0 to 3. Since rating of ‘appetite’ and ‘weight’ is duplicated (separate items for increases and decreases), only 28 items are taken into account for the final score. The scale is sensitive to change and has good psychometric properties [21].

#### Hamilton Depression Rating Scale (HDRS)

The 17-item HDRS was administered by trained research assistants with master degrees in psychology. The HDRS [20] is a semi-structured interview designed to assess depressive symptoms over the past week. It is one of the most frequently used rating scales in depression research, and internal, inter-rater, and retest reliability estimates for the overall HDRS are good [22,23].

#### Kentucky Inventory of Mindfulness Skills (KIMS)

The Dutch translation of the 39-item KIMS was used to measure mindfulness skills [24]. The KIMS was chosen as a measure of mindfulness skills because it is suitable for novices and its subscales are sensitive to change and have been validated in clinical samples [25,26]. The KIMS consists of a total scale and four subscales: (i) The tendency to pay attention to all stimuli such as thoughts, feelings and sensations (observe); (ii) The tendency to briefly label experiences and redirect attention to present moment (describe); (iii) the tendency to fully engage in current activity with undivided attention (act with awareness) and (iv) the tendency to accept thoughts and feelings as they are while refraining from applying evaluative labels such as good or bad (accept).

#### Penn State Worry Questionnaire (PSWQ)

The Dutch version of the 16-item PSWQ was used to assess worrying [27]. The PSWQ emerged from factor analysis of a large number of items and was found to possess high internal consistency and good test–retest reliability [28].

#### Rumination on Sadness Scale (RSS)

The Dutch translation of the RSS was used [29] to measure rumination. The RSS contains 13 items comprising one factor, has an adequate test–retest stability over a 2- to 3-week period, as well as good convergent and discriminant validity [30].

#### Positive affect (PA) and Negative affect (NA)

PA and NA were assessed repeatedly in daily life, using the experience sampling method (ESM; see 17). ESM is a research procedure asking individuals to provide systematic self-reports during the waking hours of a normal week. Participants rated current emotions 10 times a day at semi-random intervals on 7-point Likert scales ranging from 1 (not at all) to 7 (very). Consistent with previous work [31,32], principal component factor analysis with oblique rotation was used to generate a factor representing positive affect (PA) and a factor representing negative affect (NA). The mood adjectives ‘happy’, ‘satisfied’, ‘strong’, ‘enthusiastic’, ‘curious’, ‘animated’ and ‘inspired’ loaded on the PA factor, while ‘down’, ‘anxious’, ‘lonely’, ‘suspicious’, ‘disappointed’, ‘insecure’ and ‘guilty’ loaded on the NA factor. Mean levels of PA and NA were then computed per participant and sampling moment.

#### Childhood Trauma Questionnaire (CTQ-SF)

The Dutch translation of the short form of the Childhood Trauma Questionnaire was used [33], a self-report inventory that provides brief, reliable, and valid screening for histories of abuse and neglect. It inquires about five types of maltreatment - emotional, physical, and sexual abuse, and emotional and physical neglect. The CTQ consists of 25 items rated on a 5-point Likert scale (1 = never true, 5 = very often true). The internal consistency of the CTQ is high, it has good test–retest reliability and the criterion validity was found to be acceptable [34].

#### Interview for Recent Life Events (IRLE)

The IRLE was used to assess the occurrence of negative life events in the last year. This self-report questionnaire covers a comprehensive range of recent life events, their timing and other important qualities. The instrument consists of 63 event-specific variables. Each event is rated for month of occurrence, independence and objective negative impact. The negative impact of recent life events are rated on a five point-scale [35].

### Procedure

All study procedures were approved by the Medical Ethics Committee of Maastricht University Medical Center (MUMC+), and all participants signed an informed consent form. Recruitment started in January 2008 and ended in February 2009, and all post-intervention assessments were completed by August 2009, when the pre-determined number of participants was reached. An initial screening of potential participants was performed by phone to check for availability during the study period and likelihood of meeting in- and exclusion criteria. A second screening included administration of the Structured Clinical Interview for DSM IV axis I (SCID I [36]) and the 17-item HDRS. Eligible participants were invited for a detailed one-on-one explanation of the study procedures, and then took part in the baseline assessment. The baseline assessment consisted of six days of ESM in their own environment, and subsequent administration of a battery of questionnaires (see measures section) as well as the HDRS interview (in the laboratory). After the baseline assessment, participants were randomized to either MBCT+TAU or TAU (allocation ratio 1:1) if they were likely to have at least 20 valid ESM assessments [17,37]. After either eight weeks of MBCT (see section “Intervention”) or an equivalent waiting time (in the TAU condition), participants again took part in six days of experience sampling, followed by administration of the HDRS and questionnaires. All participants were compensated with gift vouchers worth fifty Euros. Participants in the TAU condition were offered the opportunity to take part in MBCT training after the post-intervention assessment.

Randomization was stratified according to number of depressive episodes (≤2 MDE vs ≥3 MDE), given that, as discussed above, previous studies suggested a greater benefit for those with three or more previous episodes [14,38]. An independent researcher not involved in the project generated the randomization sequence in blocks of five (using the sequence generator on www.random.org), and wrote the randomization code into sealed numbered envelopes. After completion of all baseline assessments, the researcher allocated participants to their treatment condition based on the randomization code in the sealed envelope (opened in order of sequence).

### Intervention (MBCT)

In accordance with the protocol of Segal, Williams and Teasdale [2], trainings took place in groups of 10-15 people and consisted of eight weekly meetings lasting 2,5 hours. MBCT groups started at different times throughout the year, and test periods for TAU participants were matched to MBCT participants. Sessions included guided meditation, experiential exercises, and discussions. In addition to the weekly group sessions, participants received CDs with guided exercises and were assigned daily homework exercises (30 to 60 minutes daily). Trainings were given by psychologists, general practitioners, and one psychodiagnostic worker who were all certified mindfulness trainers working in a center specialized in mindfulness trainings (SeeTrue; http://www.mindfulness-opleiding.nl). The majority of the trainers had multiple certifications from several mindfulness training institutes. All trainers also used mindfulness in personal practice. The trainers were supervised by an experienced health care professional who himself had trained with Williams and Teasdale, the developers of MBCT [2]. All mindfulness trainers were working under the guidelines of SeeTrue, which adheres to the guidelines of the Dutch Association for Mindfulness (http://www.verenigingvoormindfulness.nl/).

### Statistical analyses

All analyses were carried out according to intention-to-treat. An exploratory but systematic approach was chosen using Sobel-Goodman mediation analyses [39–41] to examine the potential effective components of MBCT. The Sobel-Goodman mediation analysis performs a statistical test to examine whether the indirect effect of the independent variable on the dependent variable, i.e. the effect exerted via the proposed mediator, is significantly different from zero. The *sgmediation* command [42] of Stata MP package 11.2 [43] was used. First, we examined which variables mediate the total effect of MBCT on depressive symptoms. We then subdivided this effect in smaller parts, hypothesizing that changes in mindfulness skills play an important role in explaining concomitant changes in other variables. Furthermore, we hypothesized that (i) resulting changes in cognitive processes and (ii) affective experiences mediate the impact of MBCT and mindfulness skills on depression outcome.

**Figure 2:**
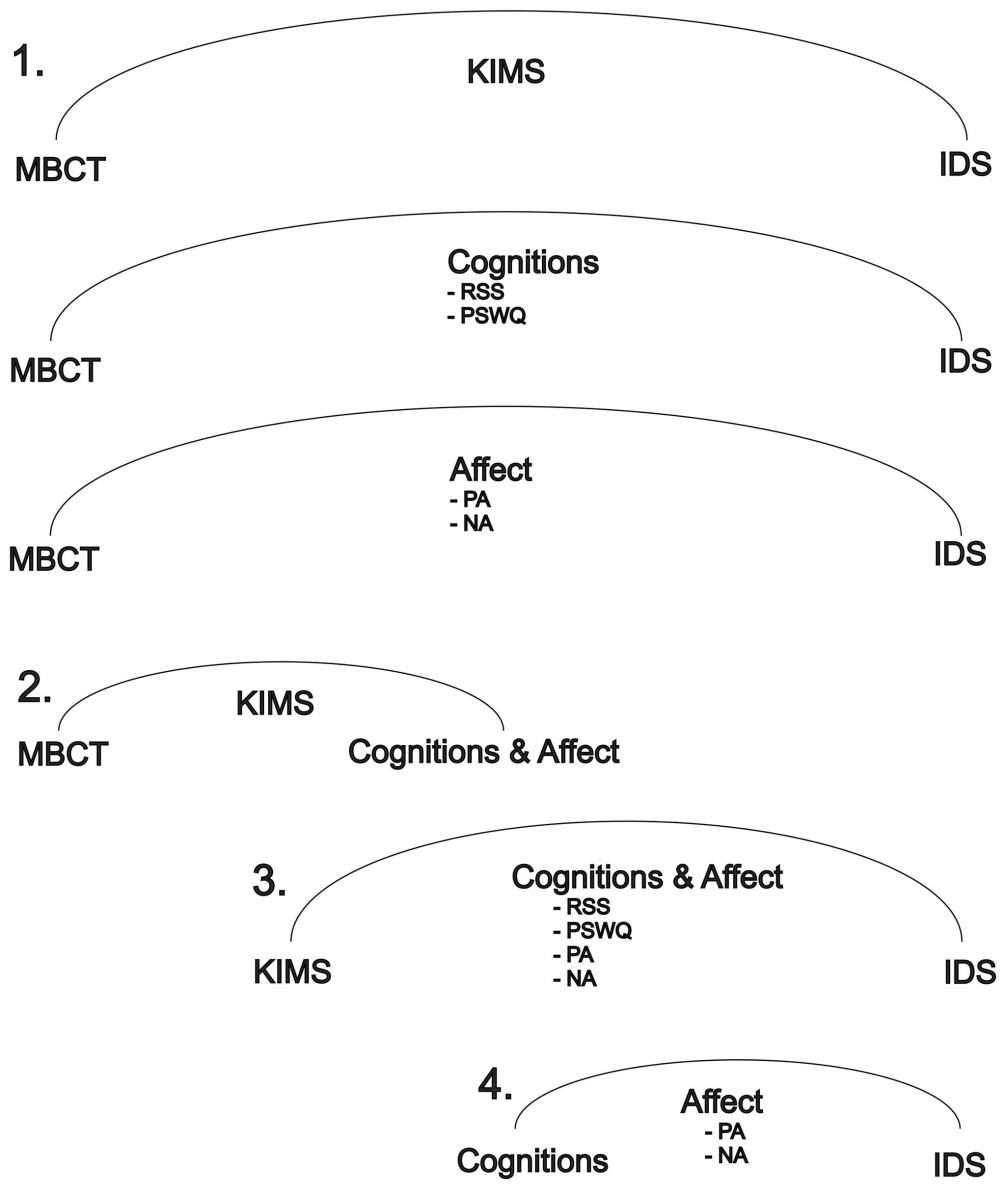
Mediation analysis pathway, for the effect of MBCT on residual symptoms of depression. N.B. All variables are change scores over time. The numbers show the four different levels of analyses.

To answer the above questions we investigated to what extent the effects of MBCT on pre-post intervention differences in depressive symptoms were mediated by changes in KIMS, PSWQ, RSS scores and momentary changes in PA and NA (level 1 in the graphical overview of the analyses in Figure 2). Next, in case of significant mediation effects of these top-level mediators, sub-effects were examined on the pathway from MBCT to depressive symptoms. First, it was examined whether, in case of significance of these top-level mediators, effects of MBCT on changes in scores of PSWQ, RSS and momentary PA and NA were mediated by changes in the KIMS scores (level 2 in Figure 2). Second, it was examined whether the effect of the KIMS scores (in case of significance) on depressive symptoms was mediated by changes in scores of the PSWQ, RSS or momentary PA and NA (level 3 in Figure 2). Finally, in case of significant mediation, we examined whether effects of the PSWQ and RSS scores on depressive symptoms were mediated by changes in momentary PA and NA (level 4 in Figure 2). This procedure was followed for the complete sample and for both subgroups (≤2 MDE and ≥3 MDE) separately.

In order to examine the independence of effects a multiple regression analysis was performed including all of the variables above as additional independent variables in the association between condition (MBCT+TAU/TAU; predictor) and reduction in depressive symptoms (dependent variable)^1^.

^1^ Since Sobel mediation analysis does not allow for additional mediators within the same analysis, multiple regression analysis was used here to obtain an indication of the independence of the mediation effects.

## Results

### Sample characteristics

A total of 130 participants were included in the study (MBCT+TAU; n = 64 and TAU; n = 66). With regard to prior psychiatric history, 71 of the participants had experienced ≤ 2 MDE, and 59 had experienced ≥ 3 MDE. Table 1 shows the demographic characteristics at baseline stratified by intervention group, and prior number of depressive episodes. There were no significant differences in baseline characteristics between the MBCT+TAU and the TAU groups. Second, there were no significant differences in baseline characteristics between the two subgroups (≤ 2 MDE vs ≥ 3 MDE). However, stratification by MBCT+TAU and TAU revealed some group differences. Within the subgroup with patients who experienced ≤ 2 MDE, more women than men entered the MBCT+TAU group compared to the TAU group. Also, for the ≤ 2 MDE group baseline IDS-SR^30^ scores were lower in the MBCT+TAU group than the TAU group, while the opposite was the case for the ≥ 3 MDE group. Childhood trauma was significantly greater in the ≥ 3 MDE group than the group with ≤ 2 MDE. There was no difference in the presence of recent negative life events between the groups.

**Table 1:** Baseline demographic and clinical characteristics.

	**Total Group (n = 130)**		**≤2 MDE (n = 71)**		**≥3 MDE (n = 59)**		**≤2 MDE vs ≥3 MDE^1^**
	MBCT	TAU	MBCT	TAU	MBCT	TAU	
Baseline characteristics; ratio (x/n)	(n = 64)	(n = 66)	(n = 35)	(n = 36)	(n = 29)	(n = 30)	
Female gender	50 (78.1%)	48 (72.7%)	29 (82.9%)	21 (58.3%)	21 (72.4%)	27 (90.0%)	NS
Psychological Treatment	13 (20.3%)	9 (13.6%)	7 (20.0%)	5 (13.9%)	6 (20.7%)	4 (13.3%)	NS
Pharmacological Treatment	33 (51.6%)	41 (62.1%)	14 (40.0%)	22 (61.1%)	19 (65.5%)	19 (63.3%)	NS
Baseline characteristics; mean (SD)							
Age	44.6 (9.7)	43.2 (9.5)	42.5 (10.6)	43.1 (8.8)	47.1 (8.0)	43.4 (10.4)	NS
Depression (HDRS)	10.3 (3.7)	10.2 (3.6)	9.6 (3.2)	10.5 (3.7)	11.1 (4.1)	9.9 (3.4)	NS
Depression (IDS-SR^30^)	22.4 (10.7)	22.5 (8.7)	19.3 (9.4)	23.8 (8.8)*	26.1 (11.2)	21.0 (8.4)*	NS
Rumination (RSS)	42.2 (9.7)	40.8 (9.7)	41.1 (9.6)	41.0 (9.6)	43.4 (10.0)	40.5 (10.0)	NS
Worry (PSWQ)	59.7 (10.9)	59.7 (10.1)	58.9 (10.3)	59.8 (11.2)	60.6 (11.8)	59.6 (8.7)	NS
Positive affect (PA)	3.6 (0.8)	3.8 (0.8)	3.8 (0.8)	3.8 (0.8)	3.4 (0.8)	3.8 (0.7)	NS
Negative affect (NA)	2.0 (0.8)	2.1 (0.8)	2.0 (0.8)	2.2 (0.8)	2.2 (0.8)	1.9 (0.6)	NS
Mindfulness Skills (KIMS)	120.0 (16.9)	121.2 (16.3)	120.6 (16.8)	122.2 (14.3)	119.3 (17.2)	120.0 (18.6)	NS
- Observe	40.0 (7.4)	40.1 (7.5)	39.6 (7.0)	41.0 (7.0)	40.1 (7.9)	39.1 (8.1)	NS
- Describe	27.1 (6.4)	26.5 (6.7)	27.3 (7.0)	26.6 (6.4)	26.8 (5.6)	26.5 (7.2)	NS
- Act with Awareness	26.1 (5.7)	26.0 (6.0)	26.3 (4.8)	26.0 (5.1)	25.9 (6.7)	26.0 (7.0)	NS
- Accept without Judgment	27.0 (7.5)	28.6 (7.3)	27.4 (6.7)	28.7 (7.1)	26.6 (8.4)	28.4 (7.6)	NS
Negative Life Events (IRLE)	3.1 (2.4)	3.2 (2.6)	2.8 (2.6)	3.4 (3.0)	3.4 (2.2)	3.0 (2.2)	NS
Early adversity (CTQ-SF)	45.2 (16.4)	44.4 (14.1)	39.3 (12.8)	41.2 (11.8)	50.5 (13.2)	50.3 (19.8)	**

Note: Subscript indicates significance levels in the comparison of MBCT vs TAU, *p < 0.05, **p < 0.001.

HDRS, Hamilton Depression Rating Scale; IDS-SR^30^, Inventory of Depressive Symptoms Self-Rating; RSS, Rumination on Sadness Scale; PSWQ, Penn State Worry Questionnaire; PA, Positive affect; NA, negative affect; KIMS, Kentucky Inventory of Mindfulness Skills; IRLE, Interview for Recent Life Events; CTQ-SF, Childhood Trauma Questionnaire Short Form

1 Significant values in this column reflect significant differences over both the MBCT and TAU group between individuals with ≤2 or ≥3previous depressive episodes. NS = Nonsignificant

Table 2 describes the change scores (post-intervention – pre-intervention) of depressive symptoms, rumination, worrying, affective changes and mindfulness skills separately per treatment group and stratified per subgroup (≤ 2 MDE vs ≥ 3 MDE).

**Table 2:** Pre-post treatment change scores of residual depressive symptoms, rumination, worrying, positive & negative affect and mindfulness skills, separately per treatment group and stratified per subgroup (≤2 or ≥3 previous depressive episodes).

	**Total Group (n = 130)**		**≤ 2 MDE (n = 71)**		**≥ 3 MDE (n = 59)**	
Pre-post intervention period change in mean, (SD)	MBCT	TAU	MBCT	TAU	MBCT	TAU
HDRS	-3.2 (4.7)	- 0.5 (4.3)**	-3.6 (3.9)	- 0.6 (4.2)**	-2.6 (5.5)	-0.4 (4.5)
IDS-SR^30^	-7.7 (8.8)	- 3.1 (8.4)**	-7.9 (8.2)	- 3.4 (8.5)*	-7.6 (9.6)	- 2.7 (8.4)*
RSS	-7.8 (8.5)	- 2.7 (7.7)**	-8.1 (8.4)	- 1.9 (6.9)**	-7.5 (8.8)	-3.7 (8.6)
PSWQ	-9.1 (8.0)	- 3.2 (8.0)**	-9.3 (7.3)	- 2.4 (7.7)**	-8.9 (6.6)	- 4.2 (8.2)*
PA	0.4 (0.7)	- 0.1 (0.6)**	0.4 (0.7)	<-0.1 (0.8)*	0.4 (0.7)	- 0.1 (0.4)**
NA	-0.3 (0.5)	<-0.1 (0.6)**	-0.3 (0.5)	<0.1 (0.8)	-0.4 (0.6)	<-0.1 (0.3)*
KIMS	14.8 (16.8)	3.1 (9.0)**	14.8 (16.8)	2.6 (7.8)**	14.8 (17.1)	3.7 (10.4)**
- Observe	4.5 (7.3)	<-0.1 (4.5)**	4.4 (7.2)	- 0.3 (4.2)**	4.6 (7.6)	0.2 (4.9)*
- Describe	1.9 (4.0)	0.7 (4.1)	1.3 (3.6)	0.8 (4.4)	2.6 (4.4)	0.5 (3.8)
- Aware	4.3 (6.6)	0.6 (3.5)**	4.9 (6.8)	0.9 (2.9)**	3.5 (6.4)	0.2 (4.2)*
- Accept	4.2 (5.5)	2.0 (4.4)*	4.3 (5.4)	1.3 (4.4)*	4.1 (5.7)	2.8 (4.4)

Note^1^: Post-intervention measures were taken 8 weeks after the pre-intervention measures.

Note^2^: Subscript indicates significance levels in the comparison of MBCT vs TAU * *p* < 0.05, **p < 0.001. HDRS, Hamilton Depression Rating Scale; IDS-SR^30^, Inventory of Depressive Symptoms Self-Rating; RSS, Rumination on Sadness Scale; PSWQ, Penn State Worry Questionnaire; PA, Positive affect; NA, negative affect; KIMS, Kentucky Inventory of Mindfulness Skills.

### Mediation analyses

**Figure 3:**
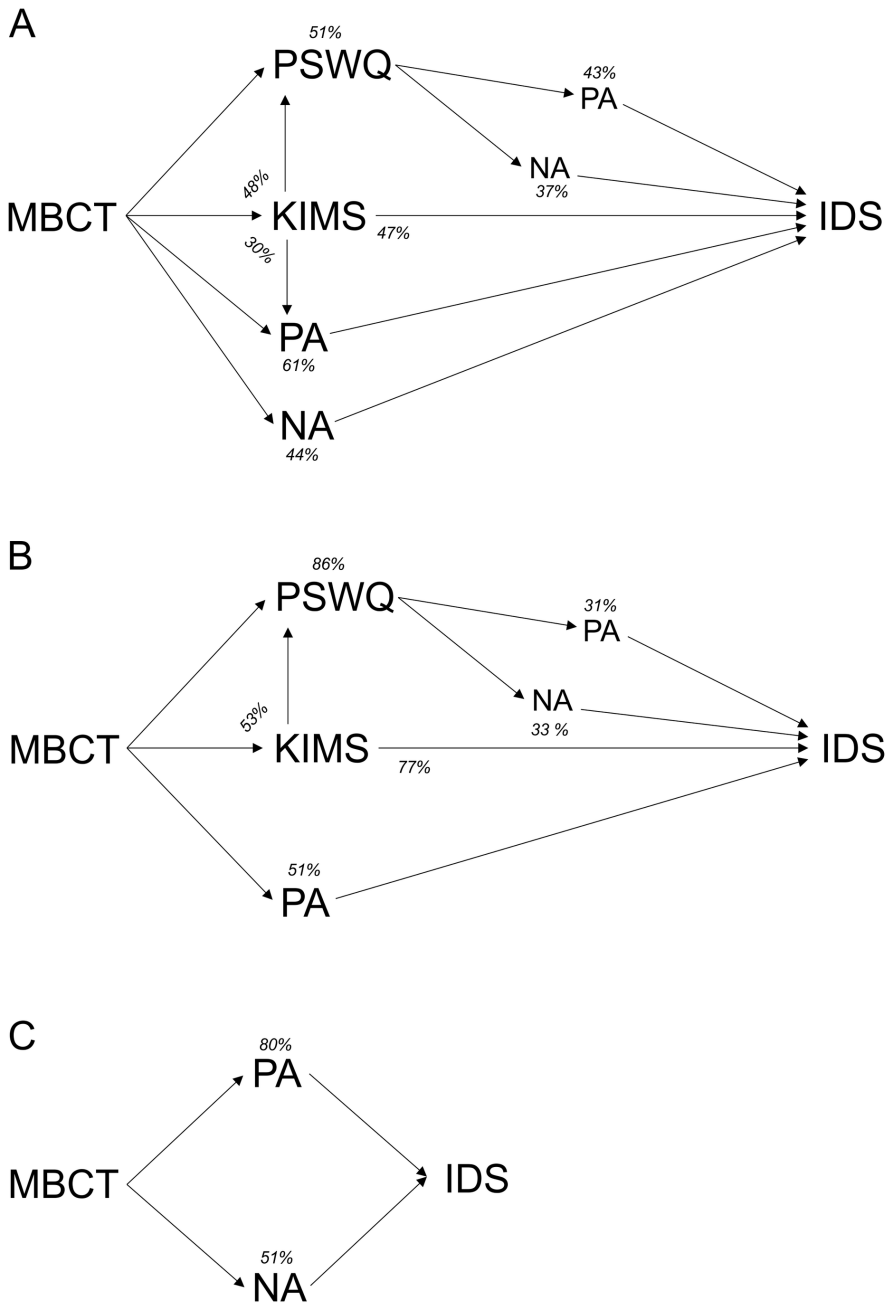
Graphical display of the resulting significant mediations for the total group and the two subgroups (≤2 MDE & ≥3MDE). **A** Mediating mechanisms MBCT for total group. **B** Mediating mechanisms MBCT for subgroup two or less depressions. **C** Mediating mechanisms MBCT for subgroup three or more depressions. The numbers in the figure show the percentage of mediation (indirect effect) of that mediator on the next phenotype. The figure can be conceived as consisting of mediation-triangles, where on top of the triangle the percentage of mediation is shown. For example in Figure 2a, the effect of MBCT on IDS-SR^30^ is mediated for 51% by PSWQ (and the effect of PSWQ on IDS-SR^30^, is again mediated by PA & NA). Also, the effect of MBCT on PSWQ is for 48% mediated by KIMS. The reported mediations do not account for a unique effect, but overlap with each other, reflecting a series of separate regressions (one for each triangle).

The effect of the MBCT intervention on depressive symptoms in the total group was significantly mediated by changes in the total KIMS score (Proportional effect = 46.8, *p* = 0.01), and specifically by changes in the subscale “acceptance” (Prop. effect = 25.2, *p* = 0.04). The effect of the total KIMS score on depressive symptoms, in turn, was mediated by changes in the PSWQ score (Prop. effect = 44.5, *p* < 0.001) and changes in PA (Prop. effect = 48.3, *p* < 0.001), but not NA. Finally, the effect of the PSWQ score on depressive symptoms, was mediated by changes in PA (Prop. effect = 43.5, *p* < 0.001) and NA (Prop. effect = 37.5, *p* < 0.001). The RSS score was not a significant mediator in this model. Table 3 shows in detail the proportional effect explained by the mediators and the significance of the effects of the tested mediation analyses. Figure 3a displays a graphical representation of the significant findings. The numbers in Figure 3a show the percentage of mediation (indirect effect) of that particular mediator on its dependent variable. The reported mediations do not account for a unique effect, but overlap with each other, as they reflect separate regression analyses. Change in positive affect was the largest mediator (61%) of the effect of MBCT on depressive symptoms.

**Table 3:** Sobel-mediation analyses.

	**Total Group (n = 130)**		**≤ 2 MDE (n = 71)**		**≥ 3 MDE (n = 59)**	
(IV – MV-DV ^a^)	Prop. effect^b^	Significance (p)	Prop. effect^b^	Significance (p)	Prop. effect^b^	Significance (p)
Level 1 (Possible mediators of effect of MBCT on depression)						
MBCT-KIMS - Depression	46.8	0.005	76.7	0.005	20.1	0.326
MBCT - Observe - Depression	16.5	0.197	39.0	0.064	-3.4	0.842
MBCT - Describe - Depression	12.9	0.154	3.3	0.657	31.3	0.119
MBCT - Aware - Depression	18.1	0.144	41.2	0.051	-2.8	0.854
MBCT - Accept - Depression	25.2	0.038	48.2	0.033	8.8	0.428
MBCT - RSS - Depression	17.4	0.127	34.4	0.094	6.0	0.604
MBCT-PSWQ - Depression	51.0	0.002	85.6	0.003	25.4	0.158
MBCT - PA - Depression	60.9	<0.001	50.7	0.038	80.1	0.007
MBCT - NA - Depression	44.4	0.009	39.9	0.112	51.1	0.036
Level 2 (KIMS as possible mediator of effect of MBCT on rumination, worry, and affect)						
MBCT-KIMS - RSS	38.1	0.007	42.2	0.013	33.4	0.202
MBCT-KIMS - PSWQ	47.8	<0.001	53.3	0.002	41.7	0.069
MBCT-KIMS - PA	29.8	0.018	41.5	0.083	20.8	0.108
MBCT-KIMS - NA	25.5	0.120	38.9	0.231	16.3	0.306
Level 3 (Rumination, worry, and affect as possible mediators of effect of KIMS on depression)						
KIMS - RSS - DepressionLevel 3 (Rumination, worry, and affect as possible mediators of effect of KIMS on depression)	9.8	0.283	9.0	0.432	8.5	0.590
KIMS-PSWQ - DepressionKIMS - RSS - Depression	44.59.8	0.0020.283	48.79.0	0.0040.432	41.88.5	0.1160.590
KIMS - PA - DepressionKIMS - PSWQ - Depression	48.344.5	<0.0010.002	29.548.7	0.0230.004	99.641.8	0.0130.116
KIMS - NA - DepressionKIMS - PA - Depression	32.948.3	0.014<0.001	23.829.5	0.0780.023	56.199.6	0.0870.013
KIMS - NA - Depression	32.9	0.014	23.8	0.078	56.1	0.087
Level 4 (Affect as possible mediator of effect of rumination and worry on depression)						
RSS - PA - DepressionLevel 4 (Affect as possible mediator of effect of rumination and worry on depression)	48.4	0.035	58.9	0.014	-1.0	0.986
RSS - NA - DepressionRSS - PA - Depression	58.248.4	0.0120.035	56.258.9	0.0220.014	57.0-1.0	0.2670.986
PSWQ - PA - DepressionRSS - NA - Depression	43.558.2	<0.0010.012	31.356.2	0.0310.022	35.257.0	0.1710.267
PSWQ - NA - DepressionPSWQ - PA - Depression	37.543.5	<0.001<0.001	33.431.3	0.0070.031	32.435.2	0.1670.171
PSWQ - NA - Depression	37.5	<0.001	33.4	0.007	32.4	0.167

MBCT, Mindfulness Based Cognitive Therapy; Depression, residual symptoms depression; KIMS, Kentucky Inventory of Mindfulness Skills; RSS, Rumination on Sadness Scale; PSWQ, Penn State Worry Questionnaire; PA, Positive affect; NA, Negative affect.

^a^ = IV, Independent Variable; MW, Mediating Variable; DV, Dependent Variable.

^b^ = Prop. effect: Proportional effect, proportion of the total effect that is mediated.

### Subgroup analyses: Two or fewer episodes

Results in Table 3 showed that the effect of MBCT on depressive symptoms in the subgroup with ≤ 2 MDE was mediated by changes in the total KIMS score (Prop. effect = 76.7, *p* = 0.01) and specifically by the subscales acceptance (Prop. effect = 48.2, *p* = 0.03) and awareness (Prop. effect = 41.2, *p* = 0.05). The effect of the KIMS on depressive symptoms, in turn, was mediated by changes in the PSWQ score (Prop. effect = 48.7, *p* < 0.001). Finally, the effect of PSWQ on depressive symptoms was mediated by changes in PA (Prop. effect = 31.3, *p* = 0.03) and NA (Prop. effect = 33.4, *p* = 0.01). Figure 3b shows a graphical representation of these findings. In contrast with the results for the total group, change in PSWQ was now the largest mediator (85%) of the effect of MBCT on depressive symptoms.

### Subgroup analyses: Three or more episodes

The effect of MBCT on depressive symptoms in the group with ≥ 3 MDE was mediated only by changes in PA (Prop. effect = 80.1, *p* = 0.01) and NA (Prop. effect = 51.5, *p* = 0.04) (Table 3). In contrast with the subgroup with ≤ 2 MDE, the KIMS and the PSWQ did not contribute to the effect of MCBT on depressive symptoms in this model. A graphical representation of these findings is displayed in Figure 3c. Eighty percent of the effect of MBCT on depression was mediated by changes in PA^2^.

^2^ Similar results were found for the total group and subsamples when the HDRS instead of the IDS SR30 was used as outcome measure (not described here).

### Independence of effects

A multiple regression analysis was performed to examine the independence of the mediators above. In the total group only the effects of NA and PA on reduction in depressive symptoms remained significant in the analysis. In both the group with ≤ 2 depressive episodes and in the group with ≥ 3 depressive episodes, only PA remained significant.

## Discussion

This study yields information about the pathway of change mediating the reduction of residual depressive symptoms during a course of MBCT. First, we replicated the finding that therapeutic effects of MBCT are mediated by changes in mindfulness skills and worry [9]. Rumination, however, did not mediate a decrease in depressive symptoms. Second, changes in positive and negative affect appeared to be important mediators for the efficacy of MBCT, and also mediated the effect of worry on depressive symptoms. Third, differential mediators may be involved in the effect of MBCT depending on prior history of MDD.

### Comparison with previous studies

In line with previous reports, changes in mindfulness skills and cognitive processes appeared important mediators in the effect of MBCT [9,15,16]. In agreement with the results reported in these studies, increases in mindfulness skills were a significant mediator in the effect of MBCT. More specifically, using the same measurement instruments, this study replicated the mediating role of change in mindfulness skills (especially mindfulness skill ‘accepting without judgment’) and worrying in the depression-reducing effect of MBCT as reported by van Aalderen and colleagues [9]. However, other results of the current study contrasted with those reported by van Aalderen and colleagues. Although rumination decreased more strongly in the MBCT+TAU than the TAU group (Table 2), we did not find a significant mediating effect of rumination in the impact on depressive symptoms. Another difference was that within the group of participants with a prior history of ≥ 3 MDE, affect predominantly mediated the effect of MBCT, whereas van Aalderen [9] found that cognitive and mindfulness processes were significant mediators.

These differences may be traced to several factors. Whereas we assessed mediation using Sobel-Goodman mediation analyses, van Aalderen and colleagues [9] used a multiple mediation model. Nevertheless, a *post-hoc* analysis using the same multiple mediation model did not change the results of the current analysis, suggesting that this is not a sufficient explanation. Another difference relates to differences in the samples that were examined in both studies. The current study only included participants with residual symptoms, whereas Van Aalderen and colleagues [9] also included participants meeting full criteria for a current episode of MDD. Mediating factors may be different for different levels of depression severity. Future work needs to address this possibility.

### Affective and cognitive factors in the mechanism of MBCT

The current study examined the role of both affective and cognitive variables in the mechanism of change of MBCT. The results suggest that PA and, to a lesser extent, NA play a substantial mediating role in the reduction of depressive symptoms during MBCT. Particularly the contribution of PA merits attention as changes in positive affect mediated 61% of the effect of MBCT on depressive symptoms. Its central role is in line with recent evidence showing that increased reward experience plays an important role in the process of remission of depressive symptomatology [17,44]. Apparently, changes in cognition and affect conjointly operate during a course of MBCT and can be hypothesized to bi-directionally impact each other.

Our findings fit within a theoretical framework on dynamic elements of emotional systems as presented by Garland and co-workers [45], describing “self-perpetuating systems energized by reciprocal causal links between the cognitive, behavioral, and somatic mechanisms through which emotions are instantiated” (page 851). These dynamics can lead to downward spirals (self-perpetuating, damaging cycles that can be triggered by negative emotions), as well as upward spirals (self-perpetuating cycles that lead to optimal functioning, which can be triggered by positive emotions). The current results highlight the potential reciprocal relations between affective states, cognitive processes, and metacognitive skills as trained during MBCT. The complex role of affective changes with their direct and indirect contributions to the reduction of depressive symptomatology is illustrated in detail by the results displayed in Table 3. The generation of PA, either as direct result of practicing MBCT exercises or indirectly through reduction of worrying, appears to be a substantial ingredient of successful MBCT. It may be that reduction in worrying promotes an upward spiral of improved affect, which in turn reduces depression-related cognitions [44–47]. This is also supported by the finding that only PA remained significant in the analyses stratified per episode group testing for the independence of the mediators. Changes in affect and especially in positive affect, which may partly result from changes in cognitive processes as worrying, thus seem to be particularly important in mediating the reduction in symptoms.

### Differential mechanisms depending on prior history of MDD

Within the group of patients with a prior history of ≤ 2 MDE, predominantly changes in cognitive and to a lesser extent affective processes mediated the effect of MBCT. However, within the group of patients with a prior history of ≥ 3 MDE, only changes in affect were significant mediators for the effect of MBCT. MBCT thus reduces depressive symptoms in both groups but, contingent on prior history, possibly through different pathways. It should be noted that although changes in cognitive processes were no significant mediators in the group of patients with a prior history of ≥ 3 MDE, it cannot be concluded that they do not play a role in symptomatic improvement in this group. It merely illustrates that changes in cognition may be of importance, but that emotional changes are the most crucial element in MBCT-related improvement.

Several reasons may explain the possible difference in mediational pathways between these groups. First, it may be explained by differences in exposure rate to proximal risk factors of which the occurrence of recent severe life-events is a plausible candidate. This seems unlikely, however, as participants in both groups reported an equal exposure to severe life-events during the last year. Second, the difference may be a result of a more advanced sensitization or scarring process in the ≥ 3 MDE group. A large body of evidence indicates that the experience of previous episodes increases the vulnerability for consecutive episodes and may result in more permanent undesirable changes in biological regulatory systems and negative patterns of information processing [46–49]. It can by speculated that depending on the level of sensitization, a more prominent role is reserved for affective changes to counteract more activated or more firmly embedded dysfunctional cognitions. Another but related aspect of prior history is age of onset of the first episode of MDD. From a developmental perspective, MDD earlier in life can have potentially more sensitization effects than a first episode of MDD at later stages in life when biological and psychological development has progressed towards adult stable levels. Regretfully, age of onset was not collected in our sample. Third, the ≥ 3 MDE group reported a greater level of exposure to childhood adversity (Table 1). This was a meaningful difference with an effect size of one SD. High rates of childhood adversity are known to be a risk factor for the development of MDD, and in addition predict a more unfavorable course [50]. Childhood adversity is thought to be an environmental risk factor leading to enduring emotional, cognitive and biological vulnerabilities for psychopathology later in life. One of these enduring effects may be manifested in the finding that emotional rather than cognitive changes were a key feature of the reduction of depressive symptoms in particularly this group. Finally, higher familial loading for affective disorders and/or differences in genetic risk profile may contribute to different sensitivity between the groups to the therapeutic effects of MBCT.

Further research is necessary to confirm these hypotheses and examine underlying mechanisms for different populations and for individuals at different stages of the illness. Findings related to differences in prior history may stimulate further development of staging and profiling models that can be used for optimal planning of treatment of individuals suffering from affective disorders [51,52].

### Strengths and limitations

This study provides new insights in the cognitive and affective factors that mediate the reduction of depressive symptoms associated with MBCT. We measured momentary affect in real-life while inclusion criteria for participants were intentionally kept broad in order to enhance generalizability. Attrition rate was low, and the analyses were intention-to treat, thus analyzing all participants who were randomized to treatment.

Some limitations should be considered. It is impossible to infer causal explanations based on our analyses because all variables were measured within the same time frame. Future studies may inform better by using prospective, multiple measurements over a complete course of MBCT to unravel in detail how affective and cognitive variables influence each other over time. Next, the current trial did not include an active control intervention. Therefore we cannot distinguish to what degree reported pathways are a direct result of MBCT or relate to non-specific intervention effects. However, it is unlikely that these non-specific effects would result in changes in mindfulness skills as observed.

Also, some participants received additional psychological or pharmacological treatment. This additional treatment was equally frequent in the MBCT group as in the control group and therefore does not confound our mediation results (see table 1). The only possibility we cannot exclude is that the observed mediation effects represent in part the synergistic effects of MBCT in combination with additional treatment. Furthermore, adherence to the MBCT protocol was not assessed formally, hence it cannot be ruled out that trainings may have differed slightly with regard to adherence. However, if this would be the case, then the currently observed mediation effects are likely conservative estimations of the true effects.

Also, patients meeting criteria for depressive disorder were excluded in this study. Therefore, the current results may not generalize to patients with current depression. Lastly, the goal of the MBCT intervention was to reduce residual symptomatology and not to prevent relapse or recurrence of MDD.

## Supporting Information

Checklist S1 **CONSORT checklist.**
(DOC)

Protocol S1 **Trial protocol.**
(DOC)
